# Differential Dopamine Receptor Occupancy Underlies L-DOPA-Induced Dyskinesia in a Rat Model of Parkinson's Disease

**DOI:** 10.1371/journal.pone.0090759

**Published:** 2014-03-10

**Authors:** Gurdal Sahin, Lachlan H. Thompson, Sonia Lavisse, Merve Ozgur, Latifa Rbah-Vidal, Frédéric Dollé, Philippe Hantraye, Deniz Kirik

**Affiliations:** 1 Brain Repair And Imaging in Neural Systems (BRAINS) Unit, Department of Experimental Medical Science, Lund University, Lund, Sweden; 2 Florey Institute of Neuroscience and Mental Health, The University of Melbourne, Victoria, Australia; 3 Commissariat à l'énergie atomique (CEA), Institut d'imagerie biomédicale (I^2^BM), Molecular Imaging Research Center (MIRCen), Fontenay aux Roses, France; 4 Commissariat à l'énergie atomique (CEA), Institut d'imagerie biomédicale (I^2^BM), Service Hospitalier Frédéric Joliot, Orsay, France; VIB & Katholieke Universiteit Leuven, Belgium

## Abstract

Dyskinesia is a major side effect of an otherwise effective L-DOPA treatment in Parkinson's patients. The prevailing view for the underlying presynaptic mechanism of L-DOPA-induced dyskinesia (LID) suggests that surges in dopamine (DA) via uncontrolled release from serotonergic terminals results in abnormally high level of extracellular striatal dopamine. Here we used high-sensitivity online microdialysis and PET imaging techniques to directly investigate DA release properties from serotonergic terminals both in the parkinsonian striatum and after neuronal transplantation in 6-OHDA lesioned rats. Although L-DOPA administration resulted in a drift in extracellular DA levels, we found no evidence for abnormally high striatal DA release from serotonin neurons. The extracellular concentration of DA remained at or below levels detected in the intact striatum. Instead, our results showed that an inefficient release pool of DA associated with low D2 receptor binding remained unchanged. Taken together, these findings suggest that differential DA receptor activation rather than excessive release could be the underlying mechanism explaining LID seen in this model. Our data have important implications for development of drugs targeting the serotonergic system to reduce DA release to manage dyskinesia in patients with Parkinson's disease.

## Introduction

Parkinson's disease (PD) is a neurodegenerative disorder affecting nearly 1% of the general population older than 60 years of age. It is characterized by loss of dopaminergic innervation in the striatum, which is responsible from motor symptoms such as bradykinesia, tremor and rigidity [1]. The most efficient treatment strategy for PD is replacement of dopamine (DA) by exogenous supplement of its precursor L-DOPA. In spite of its efficiency, long-term use of L-DOPA is associated with serious side effects consisting of motor response fluctuations and emergence of drug-induced involuntary movements, so called L-DOPA-induced dyskinesia (LID). These side effects are troublesome and limit utility of L-DOPA in patients [2]. The extent of dopaminergic neurodegeneration in the substantia nigra (SN) leading to denervation of their striatal targets is one of the major risk factors in the development of LID [3]. L-DOPA exerts its effect after conversion into DA by the aromatic amino acid decarboxylase (AADC) enzyme, which primarily occurs in residual DA terminals early in the disease. As the degeneration progresses, synthesis of DA from exogenously administered L-DOPA is gradually shifted to other cellular compartments (e.g. serotonergic neurons and non-neuronal cells). Importantly, however, these cells lack appropriate controlled release and reuptake mechanisms, therefore cannot buffer extracellular DA levels. Normally DA concentration is strictly regulated in the synaptic cleft by dopamine transporter (DAT) and the activity of presynaptic DA type 2 receptors (D2R). This helps DA to exert its effect on the post-synaptic neurons in an efficient and highly controlled manner. However, as the degeneration progresses, the number of residual dopaminergic terminals becomes insufficient to maintain this function, which results in reduced DA concentration at the synaptic sites accompanied with larger sphere of diffusion in the extracellular space [reviewed in [4]].

Postsynaptic mechanisms (i.e., status of DA receptors and second messenger signaling pathways in striatal neurons) are also known to be critical in pathophysiology of LID. The imbalance between the stimulation of D1 and D2 receptors results in a loss of synergistic activity between the direct and indirect output pathways [5], [6]. Moreover, these receptor-level modifications are caused not only by the disease itself but are also aggravated by L-DOPA treatment. Abnormal activation of striatal neurons, especially the D1R rich sub-population has been linked to alterations in transcriptional and translational factors (DARPP32, ERK1/2, CREB and δFosB), which in turn are thought to be responsible from the emergence of LID and serve as molecular markers of maladaptive plasticity in the striatum [7].

There is an increasing interest in the presynaptic mechanisms of LID. In particular, the role of the serotonergic compartment has gained considerable attention [8]–[12]. The so-called pre-synaptic serotonergic mechanism of LID stipulates that the L-DOPA precursor can be taken up by the serotonergic terminals and converted to DA, which is then stored and released from vesicles as false neurotransmitter. Serotonergic cells rely on the activity of the AADC enzyme and the vesicular monoamine transporter-2 (VMAT2) for synthesis and storage of serotonin (5HT). Thus the machinery for processing exogenously administered L-DOPA to DA is present in these cells, just as it is in dopaminergic neurons [13]–[16]. One critical distinction, however, is the release control mechanisms. Both DA and 5HT neurons retain the extracellular concentrations of their natural neurotransmitters by way of auto-receptors that can sense and regulate the amount released and uptake sites that can clear the synaptic cleft after discharge. When DA is generated in serotonergic terminals, on the other hand, this critical control mechanism becomes compromised. In support of this view, lesioning the serotonin neurons or pharmacological suppression of their activity produce near complete suppression of dyskinesia in the rat and monkey models of PD [10], [17]. Thus, it is plausible to expect that DA release from 5HT terminals would result in an uncontrolled rise in extracellular DA concentrations beyond physiological levels, which might cause worsening of dyskinesia. Despite circumstantial data to support this model, to date, there is no direct evidence showing that release from 5HT terminals indeed results in supra-physiological DA concentrations in the striatum, or abnormally high occupancy of DA receptors at the appropriate post-synaptic site.

## Materials and Methods

### Animals

A total of 250 young adult female Sprague-Dawley rats weighing between 225–250 g were obtained from Charles River (Kisslegg, Germany). The animals were housed under a 12 h light/12 h dark cycle with free access to food and water. All surgical procedures were performed according to the regulations set by the ethical committee for use of laboratory animals in Lund-Malmö region. The protocol was approved by the Malmö/Lund Committee for Animal Experiment Ethics (Permit Number: M268–08).

### Experimental design

**Figure 1:**
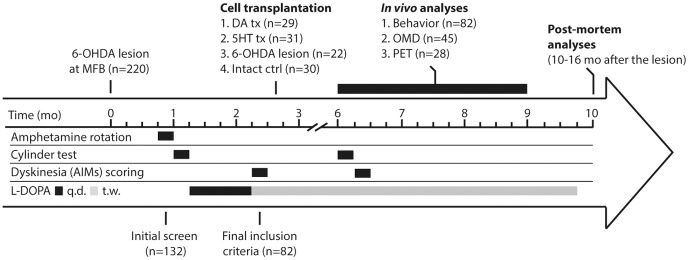
Experimental design. A total of 220 animals were lesioned with 6OHDA to remove the ascending dopamine (DA) projections unilaterally. The completeness of the lesions was confirmed with behavioral analysis and animals fulfilling the inclusion criteria were subjected to chronic daily L-DOPA treatment for induction of dyskinesia. Animals were then allocated to one of three groups balanced according to behavioral scores to receive either DA or serotonin (5HT)-rich tissue grafts or were followed as 6OHDA lesion group. Post-grafting follow up was about 7 months during which time animals underwent follow-up behavioral assessments with intervals as indicated under the study time-line. A sub-set of animals was then subjected to online microdialysis (OMD) for measurement of DA and 5HT release from the grafted neurons under baseline or after L-DOPA treatment or [^18^F]fallypride PET imaging before termination. *Post mortem* analysis included both histological and biochemical end-points (q.d.: once a day, t.w.: twice a week)-

The schematic time-line and design of the experiment is presented in Figure 1. In the beginning of the study, 220 animals received a unilateral 6OHDA lesion in the medial forebrain bundle (MFB) while 30 others were retained as intact controls. Starting 3 weeks post-lesion, the animals were screened for completeness of the lesion of the ascending dopaminergic pathway using amphetamine-induced rotational asymmetry. 60% of the rats exhibited more than six full-body turns per minute ipsilateral to the DA-lesioned side and were considered completely lesioned. These animals were then treated with daily injections of L-DOPA for 28 days to induce abnormal involuntary movements (AIMs), equivalent to peak dose dyskinesia seen in PD patients. At the end of this induction phase, 62% of animals (n = 82) exhibited stable AIMs and were retained in the study. The dyskinetic animals were then allocated into three different groups: Two groups of animals were transplanted with fetal tissue prepared as single-cell suspensions. One group received cells from the anterior segment of the ventral mesencephalon (VM) containing high numbers of dopaminergic and low numbers of serotonergic neuroblasts (referred to as DA grafts, n = 29). The second group was grafted with tissue dissected from the dorsal pontine raphe region (referred to as 5HT-grafts; n = 31), which contained high numbers of serotonergic cells but no or very few dopaminergic neuroblasts. The third group of dyskinetic rats did not receive any graft and were followed as 6OHDA lesion group (n = 22). After this point animals were kept under twice weekly injection of L-DOPA (maintenance phase) until the end of the experiment. Functional benefits of transplantation was assessed using the cylinder test for spontaneous forelimb use and AIMs test for evaluation of L-DOPA induced involuntary movements both before and three-months after the transplantation. After the completion of behavioral analyses, sub-groups of animals were subjected to one of three different microdialysis protocols for assessment of neurotransmitter release, or PET imaging to monitor the D2R occupancy during the *in vivo* follow up period. Finally, tissue were collected for either histological or biochemical end-points at termination 8–14 months after grafting (Fig. 1).

### Surgical procedures

Female Sprague Dawley rats received unilateral injections of 6OHDA (Sigma-Aldrich AB, Sweden; 3 µg/µl free base dissolved in 0.9% w/v NaCl with 0.2 mg/mL L-ascorbic acid) into the right medial forebrain bundle using a stereotaxic apparatus (Stoelting, Wood Dale, IL, USA) and 10 µL Hamilton syringe. Operations were performed under 20∶1 mixture of fentanyl citrate (Fentanyl) and medetomidin hydrochloride (Dormitor). Both drugs obtained from Apoteksbolaget, Sweden and prepared as injectable anesthetics. The antero-posterior (AP) and medio-lateral (ML) coordinates were +1.2 mm and −4.4 mm relative to bregma. The injection was made at a dorsoventral (DV) position of −7.8 mm from the surface of the dura and the tooth bar was set to −2.3 mm. The toxin was injected at a rate of 1 µL/mL and the syringe was kept in place for an additional 3 min to allow diffusion before it was slowly retracted.

For cell transplantation, the rats were anesthetized with 1–2% isofluorane mixed with 0.4 L/min O_2_ and 1 L/min N_2_O and placed in a stereotaxic frame. All transplantation procedures were performed using fetal cells dissected from embryonic day 14 rat embryos, based on a micro transplantation protocol using glass capillaries attached to a 5 µL Hamilton syringe, as described previously [10]. Tissue pieces obtained from the VM region (rich in DA neurons) or from the dorsal pontine raphe region (rich in 5HT neurons) were incubated in 0.1% trypsin/0.05% DNase in DMEM at 37°C for 20 min, and rinsed, then mechanically dissociated to a single cell suspension, centrifuged, and re-suspended to a concentration of 100,000–150,000 cells/µl for DA neuron rich grafts and 80,000–140,000 cells/µl for 5HT neuron rich grafts. The viability of the cells were >97% for both tissue preparations. A total of 3 µl from the final cell suspension was distributed over three injection tracts in the lesioned striatum (AP: +1.4, +0.5 and +0.5; ML: −2.5, −2.2, and −3.7, from bregma; tooth bar  = −2.3). Two 0.5 µl deposits (−5 to −3.5 mm below dura) were delivered along each tract.

### Behavioral tests

Three weeks after 6OHDA lesion, the animals were challenged with D-amphetamine (2.5 mg/kg, i.p.). This test was used as a screen for selection of animals for further experimentation. Animals displaying rotational asymmetry of ≥6 full body turns/min (mean over 90 min) were selected for characterization using the cylinder test and then dyskinesia induction by daily L-DOPA injections.

For assessment of forelimb use in the cylinder test, the animals were allowed to move freely in a clear glass cylinder during video recording. Mirrors were placed behind the cylinder to be able to observe all forelimb contacts on the glass wall. The videotapes were evaluated by an observer blinded to the identity of the animals and the number of left and right paw touches on the cylinder wall were counted separately for at least 20 contacts. Data are expressed as left paw touches as % of total.

In order to establish stable L-DOPA-induced AIMs (induction phase), L-DOPA methyl ester (6 mg/kg; Research Organics, Cleveland, Ohio) combined with the peripheral DOPA decarboxylase inhibitor, benserazide (10 mg/kg, Sigma-Aldrich, Sweden) was dissolved in physiological saline and administered daily to each rat as an i.p. injection for a period of 4 weeks. The evaluation of the AIMs was performed according to the rat dyskinesia scale [3]. Briefly, the animals were placed individually in transparent plastic cages with a grid lid so that the rater can visualize every movement. A researcher blinded to the identity of the animals scored each animal every 20 min following the L-DOPA injection. The AIMs were classified into three subtypes according to their topographic distribution as forelimb, orolingual, and axial dyskinesia. Locomotive dyskinesia displayed as contralateral rotations were scored separately. The severity of each AIM subtype was scored from 0 to 4 (0: no abnormal behaviors detected, 1: occasional AIMs, i.e. present less than 50% of the time; 2: frequent AIMs, i.e. present more than 50% of the time; 3: continuous AIMs, but interrupted by sensory stimuli and 4: continuous AIMs, not interrupted by sensory stimuli). Half-points were used when the behavior of the animal were clearly in between the two defined points. The data are calculated as time-integrated total scores and represented by sum of the orolingual, limb and axial subtypes.

To evaluate the effect of the 5HT receptor agonists on L-DOPA induced dyskinesia, selective 5-HT1A agonist, 8-OH-DPAT ((±)-8-hydroxy-2-dipropylaminotetralin hydrobromide; TOCRIS, Sweden), and the 5-HT1B agonist, CP-94253 (TOCRIS, Sweden) were injected subcutaneously 5 min before L-DOPA. The drugs were administered in combination at a dose of 0.1 mg/kg and 1.75 mg/kg for 8-OH-DPAT and CP-94253, respectively.

### PET imaging study

A total of 28 rats were used for PET imaging starting from 6 months after transplantation and allocated in one of 3 groups on the basis of the dyskinesia scores and cylinder tests: 6OHDA lesion group (n = 9), DA-graft (n = 8) and 5HT-graft (n = 11) groups. [^18^F]fallypride ligand was chosen for the PET imaging session because this tracer has been used to access D2/D3R occupancy in rats, baboon and human striatum [18], [19] and its ^18^F-labelling enables successive PET scan sessions. Moreover, this ligand has been shown suitable for measurement of amphetamine effects on D2/D3 ligand binding in striatum [20], [21]. L-DOPA treatment (6 mg/kg) was continued throughout the PET imaging period at the maintenance dose regimen. Each animal was imaged twice on two separate days: once under baseline conditions and a second time starting 30 min following an L-DOPA challenge (12 mg/kg). PET scans under baseline conditions were carried out 2 days after the preceding L-DOPA maintenance dose. Rats undergoing a PET scan in combination with the L-DOPA challenge did not receive the second maintenance injection within the same week.

Rats were scanned on a dedicated small animal PET scanner (MicroPET Focus 220, Siemens Medical Solutions USA, Inc.). Anaesthesia was induced by 4% isofluorane and maintained by 2 to 2.5% of isofluorane in a mixture of 100% O_2_. Before scanning, the caudal vein was catheterized with a 26-gauge catheter for intravenous injection of the [^18^F]fallypride ligand. During imaging, the head of each rat was fixed in a homemade stereotactic frame compatible with PET acquisition and the animals were maintained at 37°C using a heating blanket. Two dynamic PET scans were performed on each day of imaging. The second scan started 15 min following completion of the first one. Rats from different groups were allocated to first or second imaging slot on a given day and received either baseline or L-DOPA challenge protocol on alternative days 2 weeks apart, so that each group were represented equally as many times in first or second scan time and in either order of the two scan protocols.

Radiolabeled tracer was injected as a single bolus concomitantly with the start of PET acquisition. The injected dose was adjusted to inject similar mass of radiotracer into each separate rat (0.944±0.28 mCi; 0.486±0.198 nmol).

Rats were sacrificed the day after their last PET experiment; the brain was removed and stored at −80°C.

To study competition of [^18^F]fallypride binding in the striatal region with endogenous dopamine levels, four additional female Sprague-Dawley intact rats were scanned five times with a two-week wash-out interval between scans: one scan at baseline and four scans following increasing doses of amphetamine. Rats were subcutaneously injected with 0.1, 0.2, 1 and 2.5 mg/kg amphetamine 30 mins prior to tracer injection. These rats also underwent blocking studies (pre-saturation experiments) with a large excess of unlabeled fallypride (154.3±3.89 nmol) injected 30 minutes prior to [^18^F]fallypride in order to achieve full receptor occupancy. Displacement and receptor occupancy values (%) were calculated based on BP values measured under baseline and pre-saturation conditions.

### Radiochemistry

Ready-to-inject, >99% radiochemically pure [^18^F]fallypride (*N*-([(2S)-1-(2-propenyl)-2-pyrrolidinyl]methyl)-5-(3-[^18^F]fluoropropyl)-2,3-dimethoxybenzamide) was prepared from cyclotron-produced [^18^F]fluoride (Cyclone-18/9 cyclotron, IBA, Louvain-la-Neuve, Belgium) on the basis of already published standard conditions [22] using a tosyloxy-for-fluorine nucleophilic aliphatic substitution in a commercially available TRACERLab™ FX-FN synthesizer (GEMS, Buc, France)[23]. [^18^F]fallypride, as an ethanolic (15%) physiological saline (aq. 0.9% NaCl) solution (10–12 GBq batches, 10 mL-volume), is routinely obtained within 45 minutes starting from 30–35 GBq of [^18^F]fluoride (28–40% non-decay-corrected overall isolated yields) with specific radioactivities ranging from 222 to 333 GBq/µmol. Quality controls were performed on an aliquot of the ready-to-inject [^18^F]fallypride preparation, in compliance with the in-house quality control/assurance specifications.

### PET data analysis

Dynamic emission scans were acquired in list-mode format over 120 min. The data files were displayed as 3D sinograms with a maximum ring difference of 47 and a span of 3. The acquired data were then sorted into 31 time-frames [1*15 s, 5*30 s, 1*45 s, 6*1 min, 1*1.5 min, 4*2 min, 1*3.5 min, 5*5 min, 1*7.5 min, 6*10 min]. Finally, each emission sinogram was normalized, corrected for attenuation and radioactivity decay, and reconstructed using Fourier rebinning and 2-dimensional ordered-subsets expectation maximization (16 subsets, 4 iterations).

Time frames collected were summed to create an integrated image. In order to define volumes of interest (VOIs) using anatomical landmarks, rats underwent as well a T2-weighted MR imaging that was used for PET/MRI co-registration. To this aim, rats were placed on a 7 Tesla MR system (Varian-Agilent Technologies, USA) equipped with a gradient coil reaching 600 mT/m (120 µs rise time), a radiofrequency birdcage 1H coil for transmission, and a 4-channel surface receive coil and T2-weighted images were acquired over a total acquisition time of 9 minutes. PET/MRI co-registration was then performed using the in-house image processing software Anatomist (http://www.brainvisa.info). Striatal VOI were delineated using the microPET Data analysis software ASIPro (ASIPro VM, Siemens) and ROI drawn over 13 to 15 continuous planes.

To measure *in vivo* the fraction of [^18^F]fallypride non-specific binding, a 10-voxel-diameter spherical region of interest (voxel size of 0.47; 0.47; 0.796 mm) was drawn over the cerebellum, in a region devoid of D2R. The mean activity concentration values in all VOIs (left and right striata, cerebellum) were then calculated and plotted over time yielding regional time-activity curves. These curves were then normalized to the injected dose and body weight and expressed as standardized uptake values (SUVs).

Kinetic modelling analysis was performed using the PMOD software package (version 2.95; PMOD Technologies). D2R occupancy by the released DA in each set of experiments was estimated by calculating the distribution volume ratio (DVR) using the Logan noninvasive method [24]. This method which yields an estimate of specific binding (BP) through the relationship DVR = BP+1, assuming the existence of a reference region, such as the cerebellum, which is almost devoid of dopamine receptor and can be used to assess the pharmacokinetics of the radiotracer in the absence of a specific compartment. For each PET experiment, BP was calculated based on 9 regression points, starting after an equilibrium time of 42.5 min post-injection.

### Microdialysis experiments

The *in vivo* DOPA synthesis and DA release parameters in the striatum were assessed using a high resolution and sensitivity microdialysis protocol. For this purpose, the rats were anesthetized with 1–2% isofluorane mixed with 0.4 L/min O_2_ and 1 L/min N_2_O and placed in a stereotaxic frame. Microdialysis probes with a 3 mm membrane and 0.5 mm outer diameter (Agnthos Microdialysis, Sweden) were used. The probes were inserted into the striatum with the help of a holder and placed at AP: +1.1 mm, ML: −2.7 mm relative to bregma and DV: −5.5 mm from the dural surface. The tooth bar was set to −2.3 mm. This position corresponded to a position between the three graft deposits in the transplanted rats. The probes were connected to a syringe infusion pump (Model 100; CMA Microdialysis, Sweden) via polyethylene tubing and perfused with normal ringer solution containing 145 mM NaCl, 3 mM KCl and 1,3 mM CaCl_2_ at a constant rate of 1 µL/min. To prevent oxidization of neurotransmitters studied here, an antioxidant solution containing 1 M acetic acid, 0.27 mM EDTA, 33 mM L-cysteine, and 5 mM ascorbic acid, was mixed with the dialysate at the outlet of the probe [25].

The samples were transferred via 5 µL loops simultaneously into two flow paths and were directly analyzed at 12.5 min time bins using Alexys online monoamine analyzer HPLC system (Antec Leyden, The Netherlands) consisting of a DECADE II electrochemical detector and VT-3 electrochemical flow cell. The precise technical description of the setup has been published earlier [26]. Briefly, the outlet of the microdialysis probe was connected to a 14-port external valve that can direct the dialysate into two separate flow paths. Two different mobile phases – optimized for the detection of the respective metabolites – were used in each of the two flow paths. The first mobile phase (50 mM phosphoric acid, 8 mM NaCl, 0.1 mM EDTA, 12.5% methanol, 500 mg/L octane sulphate; pH 6.0) used for the detection of DA and 5HT ran through a 1 mm×50 mm column with 3 µm particle size (ALF-105) at a flow rate of 75 µL/min. The second mobile phase (50 mM phosphoric acid, 50 mM citric acid, 8 mM NaCl, 0.1 mM EDTA, 10% methanol, 600 mg/L octane sulphate; pH 3.2) was used for the detection of DOPA, DOPAC, HVA and 5-HIAA, which passed through a 1 mm×150 mm column with 3 µm particle size (ALF-115) at a flow rate of 100 µL/min.

We designed three different online microdialysis (OMD) protocols to address different questions relating to the hypothesis tested in this study:

#### OMD Protocol 1. KCl-induced DA and 5HT release in the striatum

This protocol was implemented to measure the releasable pool of DA and 5HT in the striatum after grafting and to determine how these pools were affected upon L-DOPA administration. The placement of the microdialysis probe was followed by one hour of equilibration period before collection of a total of 23 samples over 5 hours (i.e., each analysis point corresponded to a sampling interval of 12.5 min). Three baseline samples were collected before the dialysate was changed to a modified ringer lactate solution containing high KCl (51 mM NaCl, 100 mM KCl and 1,3 mM CaCl_2_) for a single sampling interval in order to stimulate the readily releasable pool of DA and 5HT, and then switched back to the normal ringer lactate solution. The burst of neurotransmitter release was evident in the sample with high KCl while the recovery took place during the following three sampling intervals. These 4 consecutive samples were, therefore, analyzed together to estimate the total KCl-induced release by calculating the area under the curves. After waiting for additional three time bins, 12 mg/kg L-DOPA (plus 10 mg/kg benserazide hydrochloride) was injected systemically. A second challenge with KCl was then applied during the 6^th^ time bin (62.5–80 min after L-DOPA administration) and data analyzed for four consecutive sampling intervals as described above.

#### OMD Protocol 2. Extracellular DA levels under physiological conditions

For this purpose, we designed an OMD protocol in freely moving rats where we first obtained measurements in baseline and then injected these animals with 12 mg/kg L-DOPA (plus 10 mg/kg benserazide hydrochloride)– which lead to peak dose dyskinesia that lasted for about 2 hours – and continued sampling the extracellular DA levels during this time without any other intervention or drug treatment.

#### OMD Protocol 3. Extracellular DA levels after blockade of the DAT by nomifensine

This protocol was performed in anesthetized animals, as in Protocol 1. In this experiment, measurements in baseline conditions were followed by 12 mg/kg L-DOPA (plus 10 mg/kg benserazide hydrochloride) injection, and 75 min later, the dialysis solution was switched to modified ringer lactate containing 25 µM of nomifensine and samples were analyzed for an additional 2 hours.

### *In vitro* dopamine receptor binding assay

Two groups of rats (DA denervated rats, n = 12; intact rats, n = 12) were treated with L-DOPA+benserazide (n = 7 and 7 for the two groups, respectively) or processed as non-injected controls (n = 5 per group). Sixty minutes later the animals were decapitated and striatum was dissected by removing striatum from the surrounding tissue. The dissected tissue were kept on dry ice and stored at −80°C until further use. On the day of analysis, the samples were homogenized using ultrasonic disintegrator in ice-cold assay buffer (50 mM Tris-HCl, 120 mM NaCl, 1 mM EDTA, 5 mM KCl, 1.5 mM CaCl_2_, 4 mM MgCl_2_, pH 7.4). The homogenate was diluted in ice-cold buffer and dispensed into two 96 well plates (MultiScreen_HTS_ FB, membrane pore size, 1.0/0.65 µm durapore, opaque, Millipore). One of the plates was used for D1R binding assay while the other one was used for D2R assay. A total of 50 and 350 µg of tissue lysate was used for each well in D1R and D2R binding assays, respectively. Two different radioactive ligands ([^3^H]SCH23390 for D1R and [^3^H]raclopride for D2R, PerkinElmer) were used at eight different concentrations (ranging between 0.5–15 or 1–30 nM) for each assay. In order to determine the non-specific binding, 100 mM SCH23390 or 300 mM haloperidol was used for each concentration in the two assays, where the unlabeled compound was added to the homogenate 30 min prior to the incubation with the tritiated ligand. Plates were then incubated for 2 h at room temperature. Plates were then filtered by using MultiScreen vacuum manifold (Millipore) and allowed to air-dry for 24 h. Next day, the plates were punched using a MultiScreen Punch Kit (Millipore) in order to isolate the tissue bound membranes, which were then collected individually in scintillation vials. The vials were instantly filled with LSC cocktail (Ultima Gold, PerkinElmer). Fourty-eight hours later, the radioactive decay was determined in each vial using a liquid scintillation counter (Beckman LS 6500). Data obtained in the wells treated with the cold compounds was used to measure the non-specific binding under each condition, while the corresponding wells without the unlabeled compound gave the total binding.

### Histological analysis

Rats were deeply anesthetized with 300 mg/kg sodium pentobarbital (Apoteksbolaget, Sweden) and perfused through the ascending aorta with 50 mL physiological saline at room temperature over 1 min followed by 250 mL ice-cold 4% paraformaldehyde (PFA) over 5 min. Brains were post-fixed in 4% PFA solution for 2 h before being transferred into 25% sucrose solution for cryoprotection, where they were kept until they had sunk (typically within 24–48 hrs). The brains were then sectioned in the coronal plane on a freezing microtome at a thickness of 35 µm. Sections were collected in 6 series and stored at −20°C in a phosphate buffer containing 30% glycerol and 30% ethylene glycol until further processing.

Immunohistochemical stainings were performed on free-floating sections. For this purpose, brain sections were first rinsed with potassium-phosphate buffered saline (KPBS), and then endogenous peroxidase activity was quenched by incubation in a mixture of 3% H_2_O_2_ and 10% methanol in KPBS for 30 min. After a series of rinsing steps in KPBS, non-specific binding sites were blocked by incubation in KPBS containing 0.25% Triton-X and 5% normal serum matched to the species used to raise the corresponding secondary antibody. Samples were then incubated overnight at room temperature in primary antibody solution containing 5% serum and 0.25% Triton-X. The primary antibodies used for immunohistochemical staining were as follows: mouse anti-TH (MAB318, Millipore; working dilution 1∶2000), rabbit anti-5HT (20080, Immunostar; working dilution 1∶10,000), mouse anti-SERT (MAB 1564, Millipore; working dilution 1∶1000) and goat anti-pan-FosB (SC-48X, Santa Cruz Biotechnology; working dilution 1∶15000). On the second day, the sections were rinsed in KPBS and then incubated for 1 h at room temperature in 1∶200 dilutions of appropriate biotinylated secondary antibody solutions (horse anti-mouse, goat anti-rabbit and sheep anti-goat for antibodies as appropriate; Vector Laboratories, USA). After rinsing, the sections were treated with avidin-biotin-peroxidase complex (ABC Elite kit, Vector Laboratories) and the color reaction was developed by incubation in 25 mg/mL 3,3′-diaminobenzidine and 0.005% H_2_O_2_. In order to increase the contrast in the FosB staining 2.5 mg/mL Nickel sulphate was added in the DAB solution prior to the color reaction. Sections were mounted on chrome-alum coated glass slides, dehydrated and cover-slipped with Depex mounting media (Sigma).

### Stereological analysis

The numbers of TH- and 5HT-immunoreactive cell numbers in the striatum were estimated using an unbiased stereological quantification method by employing the optical fractionator principle [27]. All quantifications were done after blinding the identity of the sections by a coding system. The borders for the region of interest was defined by using a 4x objective, whereas the actual counting was performed using a 60x Plan-Apo oil objective (Numerical aperture = 1.4) on a Nikon 80i microscope equipped with an X-Y motorized stage, a Z-axis motor and a high-precision linear encoder (Heidenhein). All three axes and the input from the digital camera that were controlled by a Computer Assisted Toolbox Software (New CAST) module in VIS software (Visiopharm A/S, Denmark), which carries out the procedure with a random start and systematic sampling routine. The sampling interval in the X-Y axis was adjusted so that at least 100 cells were counted in each grafted striatum. Upon completion of the quantification of batches, samples were moved to a database for further analysis using appropriate statistical and graphical tools. Coefficient of error attributable to the sampling was calculated according to Gundersen and Jensen [28] and values ≤0.10 were accepted.

### Image analysis

The numbers of FosB/δFosB immunoreactive cells in the striatum were analyzed on images captured using a 10x Plan-Fluor objective on a Nikon BXA microscope equipped with a Olympus DX72 camera using cellSens standard 1.5 software (Olympus Corporation). The images were captured from the striatal level corresponding to AP +1.20 relative to bregma according to the rat brain atlas of Paxinos and Watson [29]. The quantification was carried out using TIFF formatted images analyzed by using the ImageJ software (Version 1.42i, NIH). After background subtraction, the total numbers of profiles specifically labeled with FosB/δFosB were counted using the particle analysis tool. The density of immunopositive FosB/δFosB profiles was expressed as number of immunoreactive cellular profiles per mm^2^.

### Statistical analysis

The statistical analysis pertaining to the behavioral, histological microdialysis and imaging data were conducted using the SPSS statistical package for Mac version 19 (SPSS Inc., Chicago). Initial analysis was conducted using two-way ANOVA and the general linear model. When ANOVA gave significant effects this was followed by pairwise comparisons adjusted using Bonferroni correction.

Estimation of the maximum binding (Bmax) and the binding affinity (Kd) of the D1 and D2 ligands in the *in vitro* receptor binding assays were analysed using generalized nonlinear least squares using [the gnls function in] the nlme 3.1–106 [30] package in R software, version 2.15.2 [31]. The model was
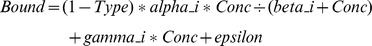



Where *Bound* is the concentration of chemically bound ligand after adding *Conc* to the sample. *Type* is an indicator being zero if only Hot ligand is added and 1 if Cold and Hot ligand is added. *Alpha_i* is the asymptotical maximum of bound ligand for treatment i (i = 1,…,4), *beta_i* is the half-saturation constant for treatment i, and *gamma_i* is the unspecific non-saturable binding in treatment i. *Epsilon* is normally distributed with variance proportional to a power function fitted value in order to obtain homoscedasticity. Comparisons of estimates between groups were adjusted for multiple comparisons using the package multcomp v 1.2–15 [32].

## Results

The precise mechanism of action by which serotonin (5HT) neurons contribute to LID in the parkinsonian striatum and how transplantation of DA neurons alleviate it while 5HT neurons worsen LID was investigated. In order to obtain results that can be unambiguously interpreted, we compared behavioral, biochemical and imaging data from intact rats and 6OHDA lesioned dyskinetic animals with that obtained from dyskinetic parkinsonian rats that received either one of two types of grafts: DA neuron-rich grafts obtained from the VM region or 5HT neuron-rich tissue obtained from the dorsal pontine raphe region of day 14 rat embryos. All animals were characterized with respect to motor behavioral deficits in the cylinder test and response to L-DOPA in the abnormal involuntary movements (AIMs) scale. Subsets of animals from each group were subjected to one of three different microdialysis protocols for assessment of extracellular DA and 5HT levels, or PET imaging to monitor dopamine D2 receptor (D2R) occupancy during the *in vivo* follow up period, and histological and biochemical end-points at termination 8–14 months after grafting (Fig. 1).

**Figure 2:**
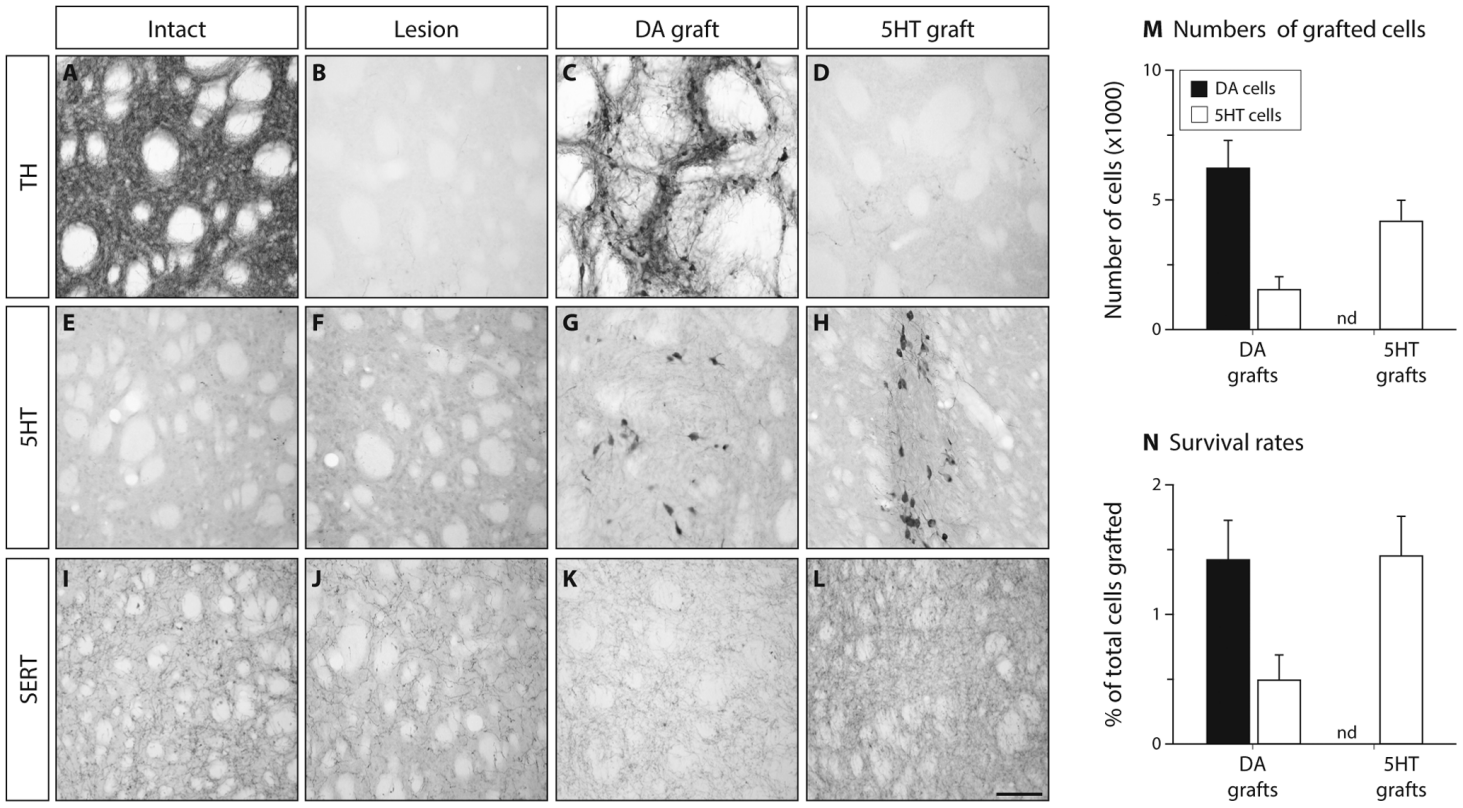
Histological characterization of the grafts. Striatal sections from fixed tissue were stained against tyrosine hydroxylase (TH), serotonin (5HT) or serotonin transporter (SERT) using immunohistochemistry. The 6OHDA lesion caused a complete lesion of the dopamine (DA) terminals in the striatum (compare A and B). VM tissue known to be rich in DA cells resulted in survival of over 6,000 TH positive cells in these long-term grafts (C, M), while 5HT grafts had no TH-positive cells (D). Immunostainings with 5HT showed numerous serotonergic cells (over 4,000) in the 5HT grafts (H), and about 1500 cells in the DA grafts (G, M). These numbers correspond to survival rates of 1.42–1.45% of the total grafted cells and illustrate excellent graft survival in both groups (N). As 5HT antibodies do not stain the serotonergic terminals well, we processed an additional set slides for SERT immunohistochemistry and confirmed that the 5HT grafts provided an intense fiber terminal network above the level seen in the intact striatum (compare I and L), whereas neither the 6OHDA lesion nor the DA grafts had a detectable effect (J, K). nd: not detected. Scale bar in panel L represents 50 µm and applies to all panels.

The 6OHDA toxin applied in the MFB caused a near complete lesion of the midbrain dopaminergic system, therefore removing the ascending projections to the striatum (compare Fig 2A and 2B), whereas the serotonergic projections from the raphe nucleus remained intact, or in some cases were partially affected, as visualized by immunohistochemical staining of the serotonergic axon terminals using antibodies against 5HT (Fig 2E, F) or the serotonin transporter (SERT; Fig 2.I, J). Transplantation of VM cells into the striatum gived rise to a new innervation source as numerous dopaminergic neurons survived and re-innervated the depleted host striatum (Fig 2C). These grafts contained, on the average, 5515±984 TH-positive cells as well as a smaller contribution of 5HT expressing neurons (1527±475 cells; Fig 2M), constituting about 1.42 and 0.49% of the total number of cells grafted in these animals, respectively (Fig 2N). When a more caudal tissue piece was used for the grafting, however, the contribution of the DA neurons was near completely abolished (as this region does not give rise to DA neurons during embryonic development), whereas the number of 5HT neurons was increased to 4169±772 corresponding to about 1.45% of total cells grafted in this group (Fig 2G, H and quantified in M, N). Importantly, in the absence of the DA neurons, the grafted 5HT neurons formed an intense supra-normal axon terminal network in the dorsal striatum, where the serotonergic innervation would otherwise be sparse (Fig 2L, compare with I–K).

**Figure 3:**
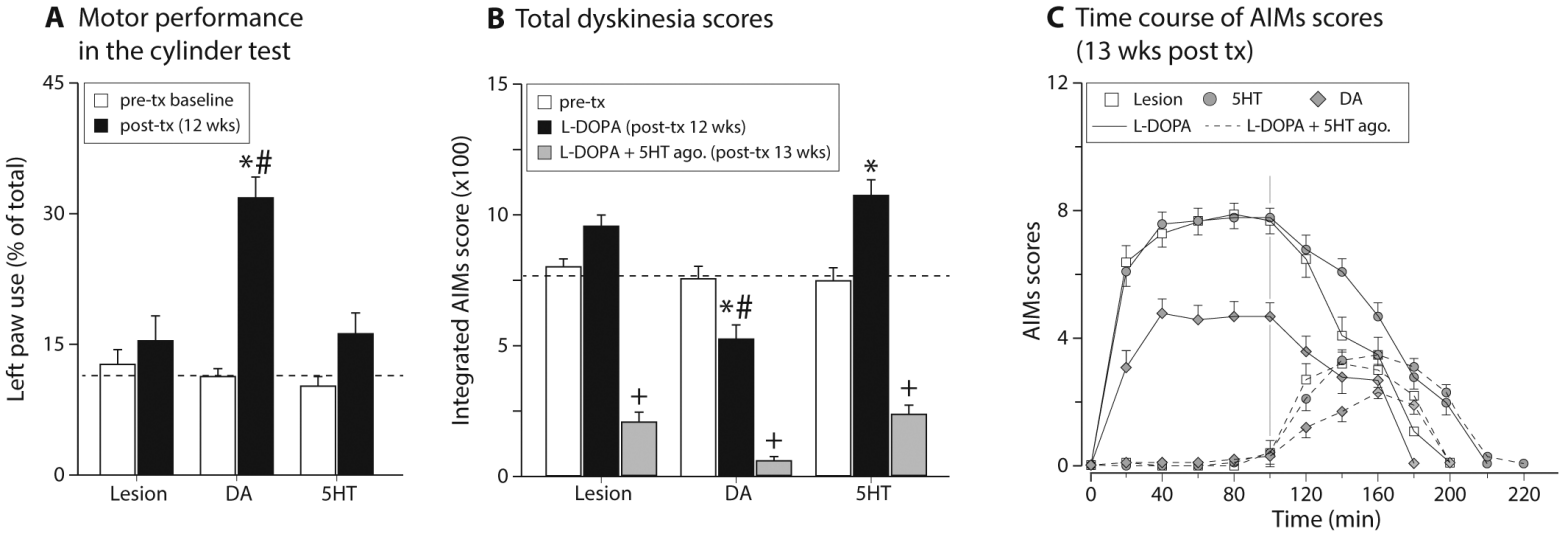
Behavioral characterization of the grafted animals. Cylinder test is a well-established spontaneous motor test based on limb-use asymmetry known to be sensitive to graft-induced functional recovery (A). All animals were severely impaired in the use of left forelimb (contralateral to the lesion side, open bars in A). 5HT grafts were functionally ineffective, whereas DA grafts ameliorated the limb use deficit significantly at 12 weeks after grafting (Two-way ANOVA F (5,163) = 16.75, p<0.001; followed by pairwise comparison adjusted using Bonferroni, p<0.0083). The animals were then challenged with L-DOPA to assess if the grafts were able to modulate the dyskinesia that were established prior to transplantation (B; two-way ANOVA F (8,245) = 63.48, p = <0.001; followed by pairwise comparison adjusted using Bonferroni, p<0.0033). DA-cell rich grafts reduced the dyskinesia, while 5HT cells were ineffective or even aggravated the dyskinesia. Co-treatment of the animals with 5HT receptor 1A and 1B agonists (0.1 mg/kg 8-OH-DPAT and 1.75 mg/kg CP-94253) reduced the dyskinesia significantly in all groups (B). The residual abnormal movements were primarily due to differential duration of action of L-DOPA and the agonists (C). tx: transplantation, *: different from pre-tx baseline; +: different from post-tx 12 wks; #: different from 6OHDA lesion and 5HT groups.

**Figure 4:**
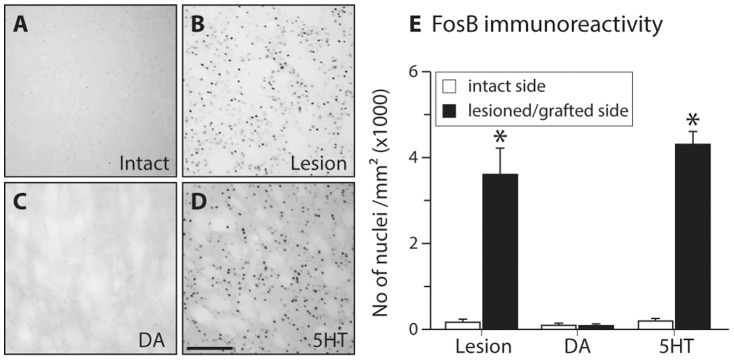
Analysis of FosB induction. The numbers of FosB/δFosB -positive cells in the striatum were assessed on a single coronal section corresponding to level +1.2 mm from bregma. Digital images were taken from the dorsolateral striatum. 6OHDA lesion group and 5HT graft animals showed significant increase in the induction number of FosB/δFosB positive profiles while in DA grafts this remained very low and similar to intact striatum (Two-way ANOVA F (5,46) = 75.05, p<0.001; followed by pairwise comparison adjusted using Bonferroni, p<0.0083). *: Different from intact side and DA grafts. Scale bar in panel D represents 50 µm and applies to all panels.

The most important difference between the two types of grafts in the context of this study was the differential ability of DA neuron-rich grafts to alleviate both the motor deficits induced by 6OHDA lesion as well as the dyskinesia induced by pulsatile administration of L-DOPA in the lesioned animals. Here, we documented the functional effects of the DA grafts by assessing forelimb use in the cylinder test. As expected, the 6OHDA lesion group displayed a deficit in use of the left (affected) forelimb, which remained unchanged in the follow-up period. There was no change in cylinder test performance after 5HT grafts, whereas the DA neurons were able to establish a functional improvement in the DA-neuron grafted animals (Fig 3A). Secondly, as the animals were subjected to chronic daily L-DOPA treatment, all animals had moderate-to-severe dyskinesia that could be seen as oro-lingual and limb hyperkinesia, axial dystonia and locomotive dyskinesia. Using a well-established rating scale [3] the L-DOPA-induced dyskinesia was quantified on three occasions; first prior to transplantation, secondly at 12 weeks post grafting when the motor improvement was documented in the cylinder test and a week later after co-injection of L-DOPA and a mixture of 5HT-1A and 1B receptor agonists (Fig 3B). We found that DA neuron rich grafts reduced dyskinesia by 30.6% while in the 5HT grafted group there was a 43.7% increase from the baseline evaluation. Thus, at 3 months after grafting, the 5HT group had 2-fold higher dyskinesia scores as compared with DA grafted animals. In all groups, dyskinesia could be substantially reduced or blocked by co-administration of the 5HT1A and 1B agonists (8-OH-DPAT and CP-94253, respectively) at doses affecting primarily the pre-synaptic auto-receptors (Gray bars in Fig 3B) [10]_ENREF_18. The residual dyskinesia in these animals was largely due to the differences between the duration of action of the agonists and L-DOPA – as the effect of the agonists waned off, abnormal movements became detectable at the end of the peak dose dyskinesia curve (Fig 3C). Of note, the reduction of dyskinesia in the DA neuron-rich grafts was accompanied with normalization of δFos-B immunoreactive nuclei in the striatum whereas 5HT neurons lacked the ability to mediate a similar effect (Fig 4). These findings confirmed that the grafts were functional and had differential effects on motor performance and response to L-DOPA.

**Figure 5:**
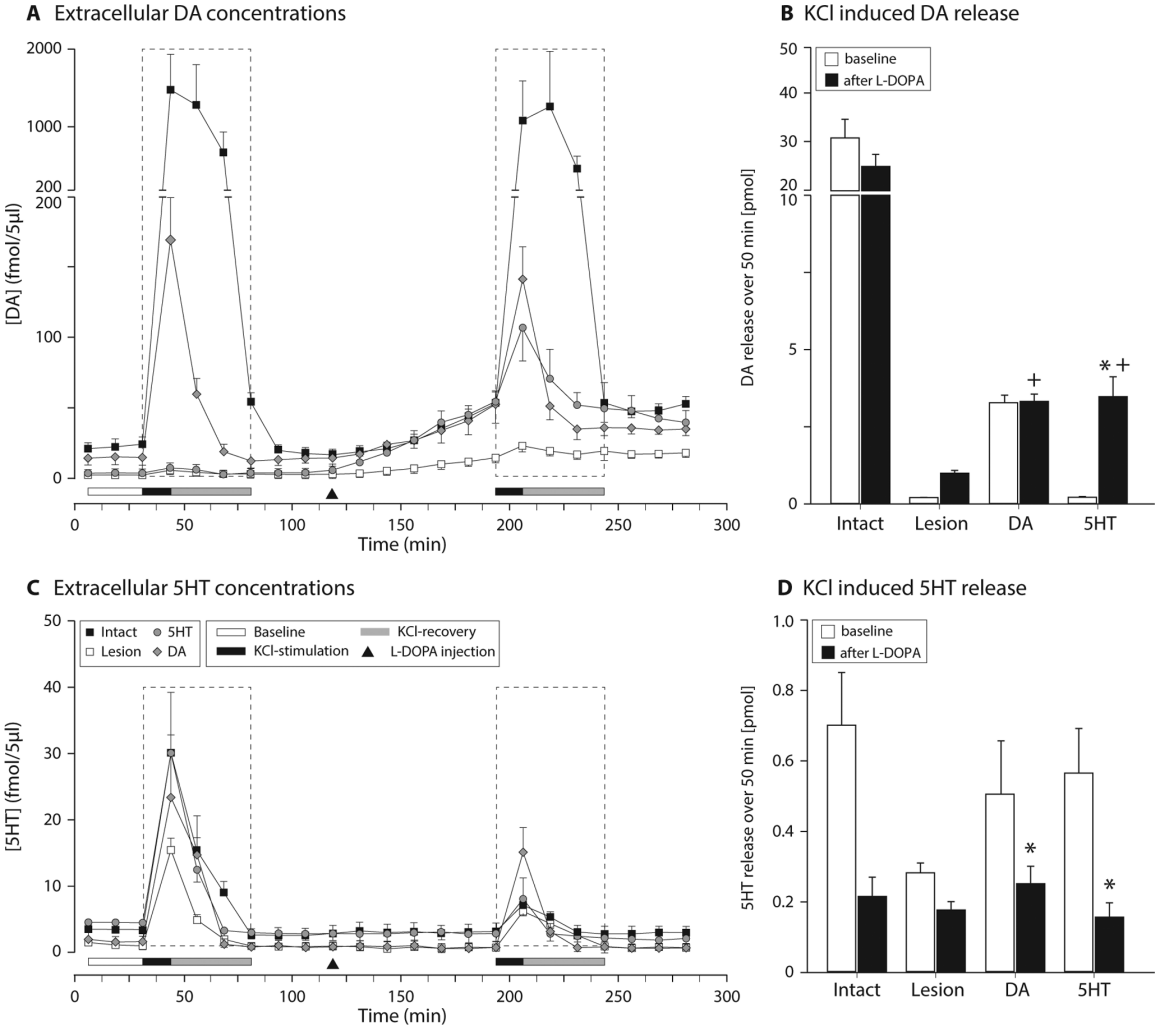
Assessment of releasable pool of DA and 5HT in the grafted animals using on-line microdialysis. Extracellular (extra-synaptic) DA levels were measured in DA depleted rats, in animals with DA and 5HT grafts as well as intact controls under anesthesia (A). This protocol included baseline assessment followed by KCl-induced release both prior to and following L-DOPA administration. Quantification of total releasable DA by KCl showed that a new pool of DA release sites emerged in the 5HT grafted animals after L-DOPA treatment, whereas this capacity was not measurable in the absence of L-DOPA (B, two-way ANOVA F (5,23) = 28.75, p<0.001; followed by pairwise comparison adjusted using Bonferroni, p<0.0083). Simultaneous measurement of 5HT release is shown in C and quantified in D. *: different from baseline; +: different from 6OHDA lesion group, comparisons are made excluding intact rats.

Investigation of whether DA released from 5HT neurons contributed to worsening of dyskinesia via a mechanism that involved fluctuations of extracellular DA concentrations with swings into the supra-physiological levels, required us to probe a series of important factors. The first step was to demonstrate that a new releasable pool of DA would emerge following systemic administration of L-DOPA in the denervated striatum, which would be increased even further in the 5HT grafted animals. For this purpose, we performed an online microdialysis (OMD) study in order to measure the extracellular levels of DA and 5HT that can be recovered by KCl stimulation (Fig 5A, C). The KCl challenges were done both under baseline conditions (i.e., prior to L-DOPA) and 60 min after injection of 12 mg/kg L-DOPA and 10 mg/kg benserazide hydrochloride which results in stable blood L-DOPA levels for at least three hours [33]. The total releasable pool of DA was estimated by calculating the area under the curve over 3 time bins (12.5 min each) following each KCl administration (Fig 5B). We found that under baseline conditions, 5HT grafts had no or minimal releasable pool of DA and was not different from the 6OHDA lesion group, whereas the DA neuron rich grafts re-constituted a clearly distinguished releasable pool, albeit at about 10% of the capacity of the intact striatum. The situation was different after the L-DOPA administration. Here, we detected a burst of DA in the 5HT-grafted animals upon KCl challenge (Fig 5). The magnitude of DA released from 5HT terminals was similar to that obtained from DA-grafted animals, suggesting that 5HT terminals became a major source of DA after L-DOPA administration in these animals (Fig. 5B). Importantly, exogenously administered L-DOPA caused no change in DA release in the intact striatum or the DA-grafted animals, suggesting that the dopaminergic terminals in these animals were able to keep the extracellular levels of DA unchanged and effectively buffer the newly synthesized DA in the tissue. Stability of the dopaminergic tone was maintained despite the fact that the 5HT terminals were involved in conversion of L-DOPA to DA in intact controls and DA-grafted rats (Fig. 5C), as KCl-induced 5HT release was diminished after L-DOPA administration in essentially all groups (change varied between 25–60%; Fig 5C, D). These observations illustrated that L-DOPA administration would involve an ectopic DA synthesis in 5HT terminals under all circumstances but that this would be effectively buffered by efficient re-uptake in the presence of a dense DA terminal network in the vicinity of the serotonergic terminals.

**Figure 6:**
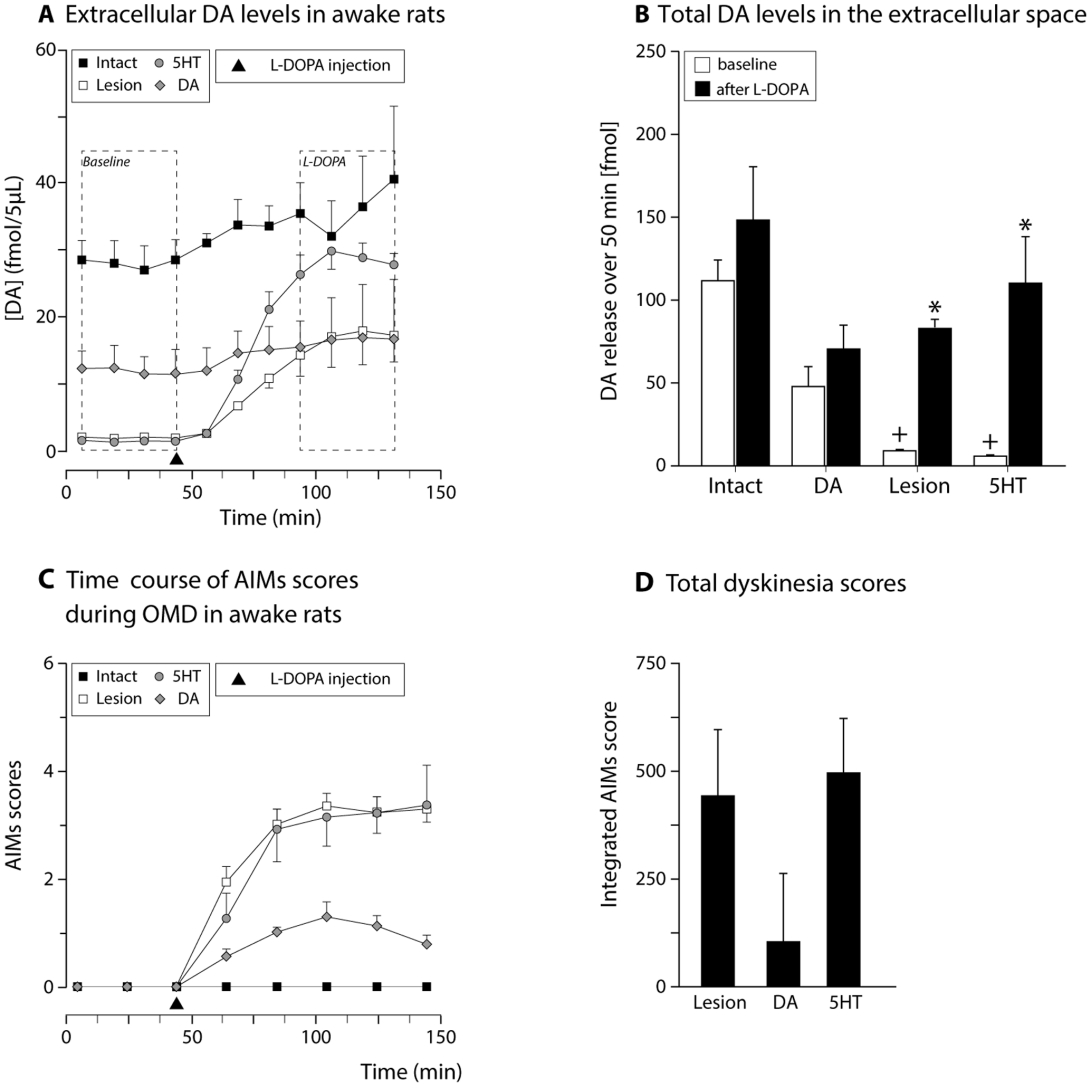
Assessment of changes in extracellular DA levels following L-DOPA injection. Animals were subjected to on-line microdialysis measurements either under baseline conditions, or following a systemic injection of 12 mg/kg L-DOPA without any perturbation of the release sites using release-inducing drugs. Time course data is shown in A, while quantification of the two phases (4 time bins in each case) is given in panel B (Two-way ANOVA, F (7,27) = 12.15, p<0.001; followed by pairwise comparison adjusted using Bonferroni, p<0.0071). Panel C illustrates the dyskinesia rating scores obtained during the OMD experiment as the animals were sampled in awake and freely moving state. Panel D shows the integrated AIMs data from this session (One-way ANOVA, F (2,10) = 13.11, p = 0.003; followed by pairwise comparison adjusted using Bonferroni, p<0.017). Note that the dyskinesia seen in lesion and 5HT groups occur in the absence of supra-normal DA levels as detected by the probe in the striatum *: Different from baseline; +: different from intact; #: different from lesion and 5HT groups.

Next, we investigated how extracellular DA levels were changed upon L-DOPA administration under physiological conditions (i.e. in the absence of KCl challenge). For this purpose, we performed OMD in awake and freely moving rats that were naïve to L-DOPA. We first obtained measurements at baseline and then injected these animals with 12 mg/kg L-DOPA – which lead to peak dose dyskinesia that lasted for about 2 hours – and continued sampling the extracellular DA levels during this time period (OMD results are shown in Fig. 6A, B, while the corresponding dyskinesia rating is given in Fig. 6C, D). The results were interesting: As expected, DA levels in the intact controls and DA-grafted animals were stable with minimal changes upon L-DOPA challenge. In the 6OHDA lesioned animals, DA levels at baseline were very low (about 8% of intact), and started to rise after L-DOPA before stabilizing at about the same level as in the DA grafted animals (Fig. 6A), despite that the 6OHDA lesion group had more severe dyskinesia while DA grafts reduced them in the transplanted group. Moreover, in the 5HT-grafted animals – where the dyskinesia was most severe– the changes in extracellular DA levels followed a similar drift as in 6OHDA lesion group, but reached levels comparable to that seen in the intact animals (Fig 6B). There was however no indication that DA released from the serotonergic terminals reached supra-physiological levels.

**Figure 7:**
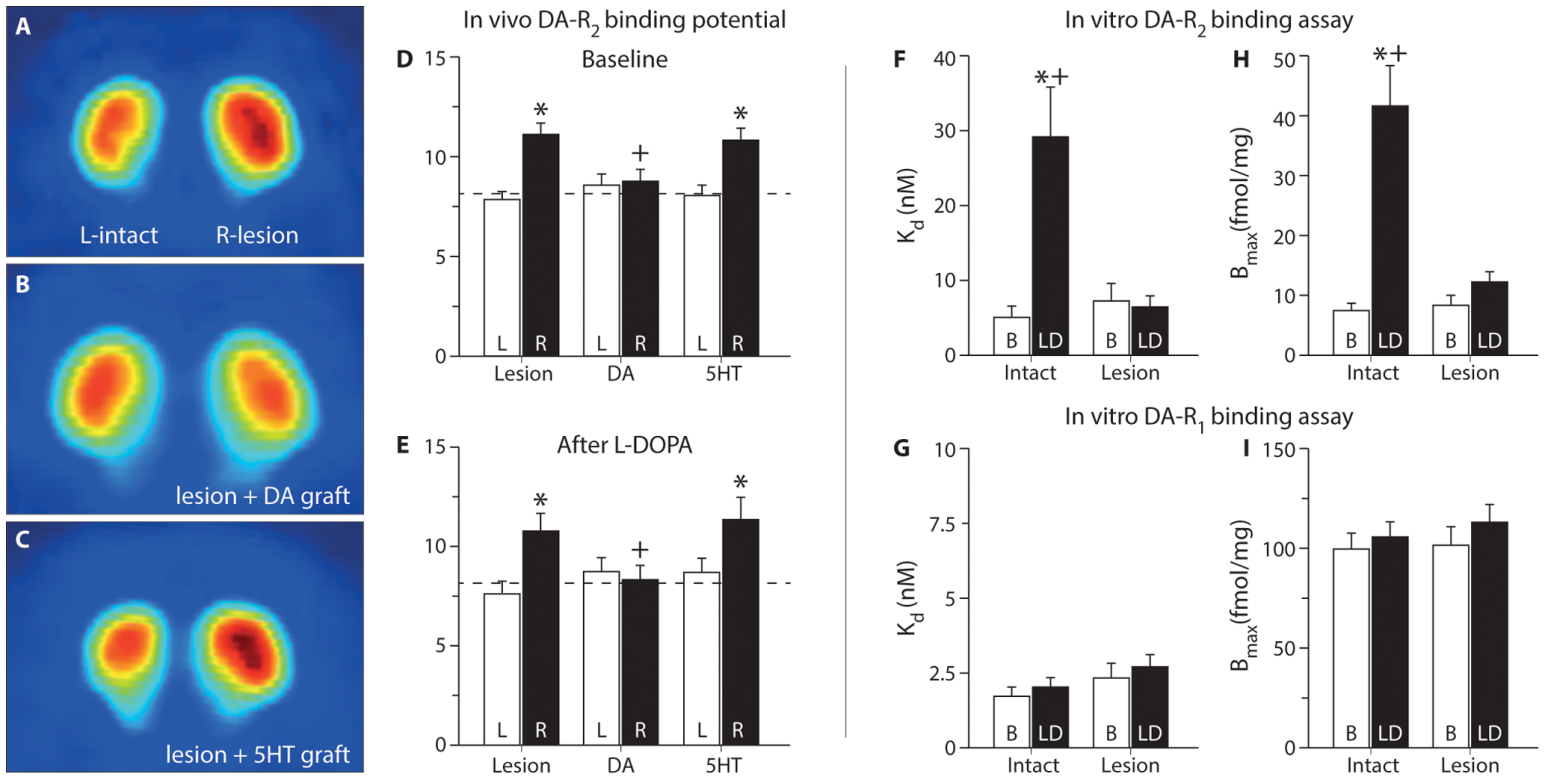
Assessment of striatal dopamine D2 receptor occupancy using positron emission tomography imaging. A subset of animals (n = 28) were subjected to two [^18^F]fallypride PET imaging experiments between 4–7 months after grafting. The first examination was done under baseline conditions whereas the second one was performed starting 30 min after L-DOPA treatment. This radioligand binds to the D2 receptors. The signal is increased when the endogenous ligand is lost, as seen in the 6OHDA lesion group under baseline conditions (A). The abnormally increased binding is completely normalized in DA grafted rats (B), but remained unchanged in the 5HT-grafted animals (C). The imaging data was quantified using Logan plots to determine the binding potential in the striatal tissue (D; two-way ANOVA F (5,55) = 6.70, p<0.001; followed by pairwise comparison adjusted using Bonferroni, p<0.008). L-DOPA injection did not result in any change in the binding potential (E; two-way ANOVA F (5,55) = 3.15, p = 0.15). *In vitro* receptor binding assay was performed for D2R (F,H) and D1R (G,I) and analyzed using a generalized non-linear model. In panel F and H, post-hoc comparisons between B and LD in the D2R in the intact brain is p<0.001 for K_d_ and <0.002 for B_max_, respectively and post-hoc comparisons between LD injected intact and lesioned brains is p<0.001 for K_d_ and <0.003 for B_max_, respectively. L: left (intact) side, R: right side, B: Baseline; LD: L-DOPA treatment, K_d_: binding affinity, B_max_: Receptor density. *: Different from intact side; +: different from 6OHDA lesion group.

To establish whether DA released from serotonergic terminals resulted in abnormal, post-synaptic activation of the striatal neurons – as a basis to induce and/or worsen dyskinesia – we investigated whether the occupancy of DA receptors upon L-DOPA administration in these animals surpassed normal physiological levels. This would be reasonable to expect based on the earlier stated hypothesis postulating that DA released from 5HT terminals would be uncontrolled and thus could exceed the physiological levels normally seen in the intact striatum [10]. Moreover, the results from the KCl-challenge experiments described above confirmed that a new pool of DA had indeed emerged after L-DOPA administration in these animals, confirming that the substrate for this abnormal release mechanism existed. Demonstration of the presence of supra-physiological DA in the 5HT grafted animals, however, required a different measurement technique than microdialysis as this method would only inform on extracellular DA that diffuses in the extra-synaptic space and is taken up by the probe, as opposed to DA levels at the specific release sites, which in turn determines the level of activation of DA receptors (i.e., their occupancy by DA). Therefore, we utilized [^18^F]fallypride PET imaging as a means to estimate occupancy of the D2R pool *in vivo* by calculating the binding potential (BP) of the ligand to the receptors in the presence and absence of L-DOPA (Fig. 7). Under baseline conditions (i.e., in the absence of L-DOPA), animals with unilateral 6OHDA lesions showed increased [^18^F]fallypride ligand binding to D2R's compared with the contralateral intact side, where dopamine from the preserved ascending projection to the striatum competes with [^18^F]fallypride at the D2R binding (Fig. 7A). The DA-neuron rich grafts completely normalized this abnormally high binding suggesting that these cells can re-constitute a normal DA signaling at the appropriate receptor sites in the living animal (Fig. 7B), while the 5HT grafts lacked this ability (Fig. 7C). We expected that the administration of L-DOPA in the 5HT-grafted animals would significantly alter the BP of [^18^F]fallypride tracer, if indeed it resulted in abnormally high DA levels in the peri-synaptic space. However, we found that the administration of L-DOPA did not result in any change in the BP obtained in these animals (compare BP values in Fig. 7D and E).

**Figure 8:**
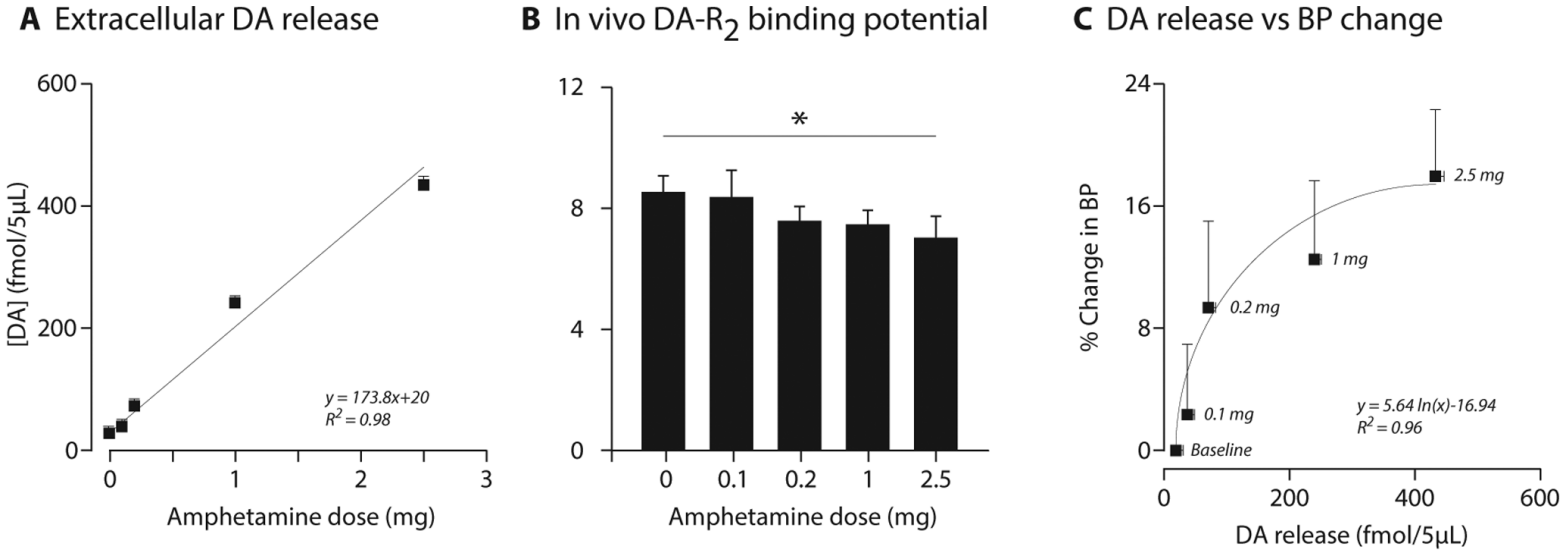
The relationship between dose of systemic amphetamine administration, DA concentrations and [^18^F]fallypride binding potential. Extracellular DA levels following different doses of amphetamine measured using online microdialysis (A). Note the tight and linear correlation between the two parameters. In animals pre-treated with the same doses of amphetamine, the binding potential (BP) for [^18^F]fallypride was reduced (B, *: effect of dose, one-way repeated ANOVA F(3,9) = 6.99, p = 0.006). The relationship between extracellular DA concentration and BP followed a logarithmic curve (C). The formulas indicated in panels A and C refer to the best fit for the respective data.

The [^18^F]fallypride PET imaging data were at odds with the expected results but supported the interpretation that DA released from 5HT terminals (both in the presence of a 5HT graft and in the DA denervated striatum) resulted in low concentrations of DA at the D2R containing sites thus a low occupancy of these receptors. In order to demonstrate the displacement of [^18^F]fallypride, we performed additional experiments where we determined the dose-response relationship between amphetamine-induced DA release and changes in [^18^F]fallypride BP. In occupancy studies using amphetamine challenge, BP values were compared to baseline values. Dose dependent decreases in [^18^F]fallypride BP (−2.36%, −9.32%, −12.48%, and −17.93%) were seen in the striatum following 0.1, 0.2, 1 and 2.5 mg/kg doses of amphetamine, corresponding to 38.3–434.3 fmols DA released in the extracellular space in intact rats, respectively (Fig. 8A–B). In addition, full saturation studies using a large excess of unlabeled fallypride injected prior to the radiotracer indicated that 88.31% of the [^18^F]fallypride in vivo binding relates to specific (displaceable) binding to dopamine receptors. Morover, up to about 20% of the receptor pool was sensitive to displacement by endogenous DA released in the extracellular space after amphetamine treatment. The two parameters (extracellular DA levels and % changes in BP) correlated with one another and suggested a logarithmic relationship (Fig. 8C), note however the data are obtained in different animals due to technical difficulty of simultaneous measurements.

To confirm the above PET imaging results obtained *in vivo*, we used the gold-standard *in vitro* receptor assays to directly determine the Kd and Bmax values for the D1 and D2 receptors. For this purpose, we analyzed the brains of a separate group of intact and 6OHDA lesioned rats killed either under baseline or 1 hr after L-DOPA injection at the peak of dyskinesia. Striatal tissue from these brains was processed for D1R and D2R receptor binding using [^3^H]SCH23390 or [^3^H]raclopride ligands (Fig. 7F–I). Comparison between baseline and post-L-DOPA conditions in the intact brain showed that the primary site of activity for the newly synthesized DA was at the D2R (Fig 7F,H), while no significant change occurred at the D1R (Fig. 7G,I). This suggested a selective activation of the D2R by DA after released from the endogenous terminals. In the lesioned striatum, where DA release is predominantly from the ectopic serotonergic terminals, the results were different as no change occurred at the D2R.

**Figure 9:**
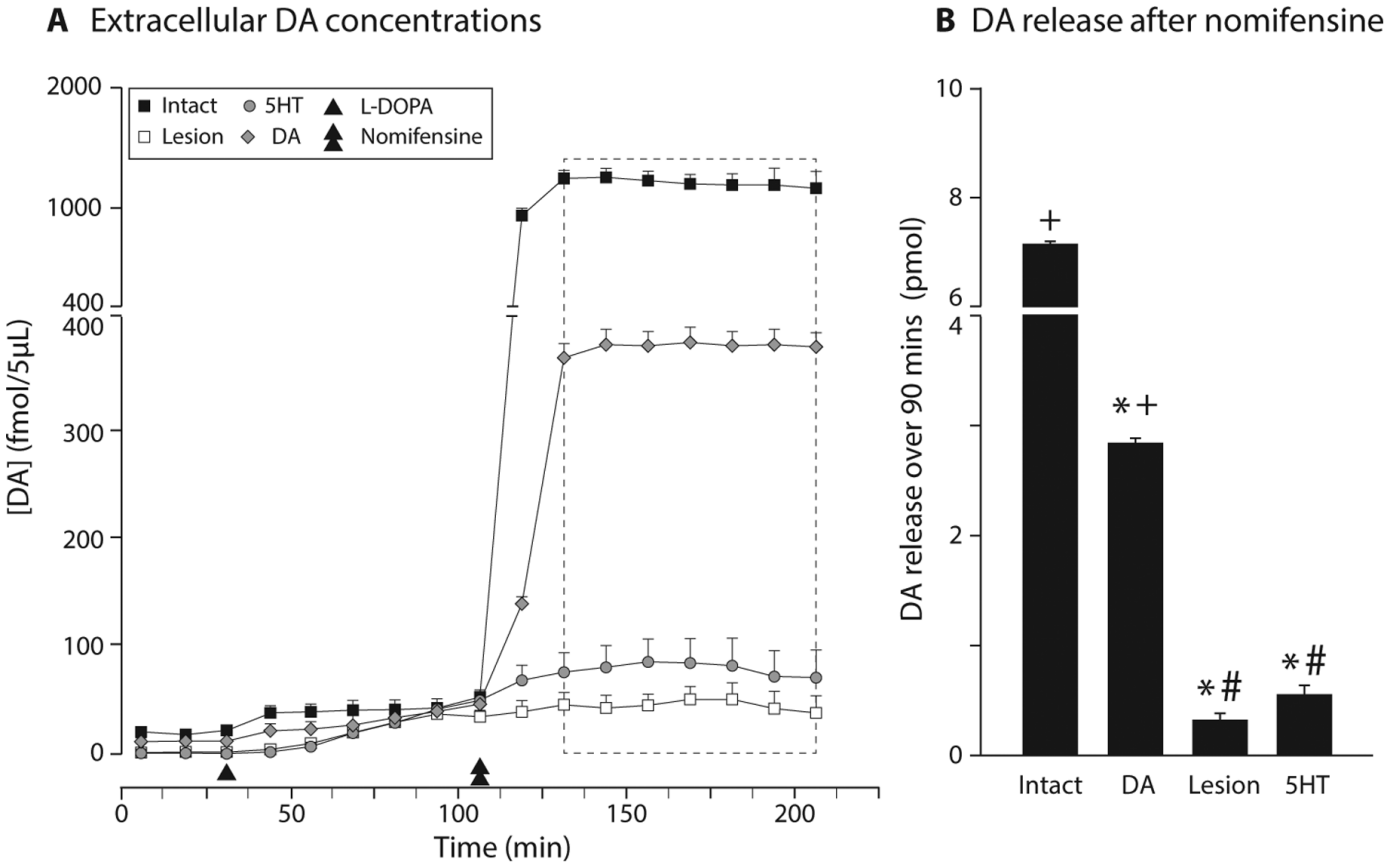
Assessment of changes in extracellular DA levels following L-DOPA injection and nomifensine treatment. Anesthetized animals were subjected to OMD measurements first under baseline for 37.5(3 time bins), and then injected systemically with 12 mg/kg L-DOPA. At 125 min, they received nomifensine (DAT blocker) using the reverse dialysis method. The time course data shown from this experiment is shown in A and quantification of the plateau phase after nomifensine (7 time bins over about 90 min) is given in panel B. (One-way ANOVA F(3,15) = 166.48, p<0.001; followed by pairwise comparison adjusted using Bonferroni, p<0.0083). *: Different from intact; +: different from lesion; #: different from DA graft.

Taken together, these findings lead us to consider an alternative hypothesis to the previously held model (see discussion). In order to further substantiate the evidence to support this hypothesis, and demonstrate the magnitude of difference in DA released from DA-neurons and that released from 5HT-neurons, we carried out a third microdialysis study where we monitored the DA captured by the microdialysis probe after blockade of DAT by nomifensine (Fig. 9). Inhibition of the re-uptake sites by nomifensine resulted in a rapid and dramatic rise in the amount of DA captured by the microdialysis probe and revealed the true difference in the amount of DA that is generated at the synaptic site in the intact striatum and re-constituted after grafting DA neurons versus the very low levels in the 6OHDA lesion group and 5HT-grafted animals (time line shown in Fig. 9A). We found that the total amounts of DA recovered in DA grafted animals and in the intact rats were 5.2–8.9, and 16.2–28.1 fold higher than the lesioned and 5HT-grafted groups, respectively (Fig. 9B).

## Discussion

DA released from serotonin neurons as a false neurotransmitter is a key factor in the induction of LID in the 6OHDA rat model of PD. However, the precise mechanism accounting for this event has not been demonstrated. The so-called pre-synaptic serotonergic mechanism of LID stipulates that an abnormally high and uncontrolled DA release might be the underlying pathophysiological event contributing to induction and maintenance of dyskinesia in animal models of PD [34], [35]. Here we designed experiments to directly test the validity of the hypothesis that upon administration of exogenous L-DOPA, DA released from serotonergic terminals reaches supra-physiological levels and whether DA released as a false neurotransmitter is associated with abnormally high occupancy of DA receptors on the striatal neurons.

For this purpose, we compared the *in vivo* DA release properties and D2R occupancy under 4 conditions: (1) intact striatum where the DA terminal network is intense and far above the endogenous 5HT terminal density; (2) lesioned striatum with near complete loss of DA innervation while the 5HT fiber terminals are at least partially retained; (3) denervated striatum grafted with 5HT neurons in which the total 5HT fiber density is enhanced beyond the sparse network present endogenously; and (4) denervated striatum transplanted with DA-neuron rich grafts that partially re-constitutes the normal DA terminal network, and in fact also contained some 5HT neurons as well.

Although the data we obtained confirmed that 5HT terminals were recruited to ectopically synthesize and store DA upon L-DOPA administration, the level of extracellular DA measured by OMD did not exceed those seen in intact animals – either in parkinsonian rats or in animals where grafts rich in 5HT neurons generated a supra-normal serotonergic terminal density in the dopamine denervated striatum. Moreover, [^18^F]fallypride PET imaging showed that the D2R occupancy in the striatum was in fact not altered in either group of animals, suggesting that DA originating from serotonergic terminals could not have contributed to a large increase in DA bound to D2R.

Exogenous L-DOPA induced a KCl-releasable pool of DA accumulating in the serotonergic terminals originating from the 5HT grafts comparable to what we measured in DA grafts suggesting that in the presence of L-DOPA, the serotonergic terminal network served as a potent source of DA comparable to that generated by conventional VM grafts. Importantly, however, enriching the DA synthesizing compartment in the denervated striatum by DA- or 5HT-grafts had distinctly different functional consequences. First, DA grafts reduced dyskinesia and improved normal motor performance, whereas 5HT grafts appeared ineffective in reducing motor impairments and worsened dyskinesia. Secondly, under baseline conditions, DA grafts reconstituted extracellular DA levels, which remained stable even after administration of L-DOPA peripherally. 5HT grafts, on the other hand, not only lacked the ability to normalize DA neurotransmission, but also failed to buffer the newly formed extracellular DA upon L-DOPA challenge as the DA levels in the striatum started to drift. The OMD measurements showed that although DA levels increased by 19 to 27-fold in lesioned animals, the peak levels reached at about 2 hours after L-DOPA administration still remained within the normal physiological range. These findings are in agreement with data reported by Lindgren and collaborators [11]. Third, while DA grafts were efficient in normalizing the BP for [^18^F]fallypride in the striatum, 5HT grafts were ineffective in mediating a similar correction even after L-DOPA administration. The latter finding, in particular, suggested that DA released from the 5HT terminals was inefficient in re-constituting dopaminergic neurotransmission, at least via the D2R mediated pathway.

In both PD patients and 6-OHDA lesioned animals, where there is a severe loss of dopaminergic terminals in the striatum, synaptic DA concentrations remain at very low levels of L-DOPA. Two characteristic features of this phenomenon might have an impact on occurrence and severity of dyskinesia. First, DA neurotransmission is re-constituted, albeit at insufficient levels, only in the presence of peripheral L-DOPA. Second, each L-DOPA treatment causes a short-term increase in DA levels exposing the striatal neurons to a transient DA-receptor stimulation. Thus, the post-synaptic neurons maintain an abnormal exposure and response to DA stimulation. The characteristics of generation of DA in the striatum might still be one of the critical factors in expression of dyskinesia in lesioned animals, as a recent study found differences in peak striatal DA concentrations between non-dyskinetic and dyskinetic rats, although data in both groups remained below levels measured in normal rats [11]. Given that we have not been able to show the presence of an excessive DA release from serotonergic terminals and that maladaptive plasticity in the striatal neurons persisted, it is plausible that the pathophysiological basis of LID in these animals relies on a transient activation of super-sensitive receptors. Stocchi and colleagues (1995) showed that the benefits of continuous dopamine stimulation were obtained despite that the blood levels of L-DOPA in these patients were higher during continuous infusion as compared with the intermittent oral administration, supporting the view that dyskinesia was probably related to the pulsatile nature of the stimulation rather than the level of L-DOPA *per se* [36].

It is notable that in the experimental conditions studied here, D2R occupancy at sub-physiological levels might be part of a mechanism that gave rise to severe dyskinetic behaviors in parkinsonian animals that have a rich serotonergic terminal network in the striatum. Therefore, another mechanism, not mutually exclusive of the aforementioned, might relate to the precise activation profile of DA receptors in the striatum. It is well known that activation of D1R provoke severe dyskinesia, while the use of D2R agonists do not cause the same side effects [6], [37]–[42]. Thus, a differential activation of the two-receptor subtypes by DA might be the cause of the substantially different behavioral outcomes. Our findings in this experiment suggest that abnormal behavioral outcomes (such as occurrence of dyskinesia) could be seen when D2R activation fails, while the D1R binding properties remain unaltered.

DA receptors (at least the D1R type) are known to be located on the soma and dendrites of striatal neurons where dopaminergic neurons do not normally make synaptic contacts [43], [44]. Our OMD data showed similar levels of DA in the extracellular space in the 5HT-grafted L-DOPA treated animals and the intact rats. Therefore, it appears that the extra-synaptic DA-receptor occupancy would be comparable in these two scenarios. This raises the possibility that activation of extra-synaptic D1R, in the absence of appropriate D2R activation, could be one of the underlying reasons for occurrence of LID, and 5HT-neuron mediated DA release might lead to this unwanted outcome.

The effect of L-DOPA on D2R occupancy has been studied using another PET ligand, [^11^C]raclopride, where the investigators found that the BP values after L-DOPA treatment was lower and this was reversed by use of selective 5-HT1A agonist, 8-OH-DPAT [45]. Although the characteristics of the [^11^C]raclopride and [^18^F]fallypride tracers are reported to be similar [46], we cannot rule out that displacement of [^11^C]raclopride would be achieved at lower levels of re-constitution of DA in the striatum. In a series of additional experiments done in healthy rats, we found that there is strong relationship between amphetamine induced DA release and % decrease in [^18^F]fallypride BP obtained by PET imaging (Fig 8). In addition, the study by Nahimi and colleagues used an acute challenge paradigm and administered high dose L-DOPA (50 mg/kg) to animals prior to imaging. The dose selected in that study would not be possible to implement in a chronic treatment paradigm like the one we used here, as it would result in self-mutilation in animals and in fact is much higher than clinically applied doses in man.

**Figure 10:**
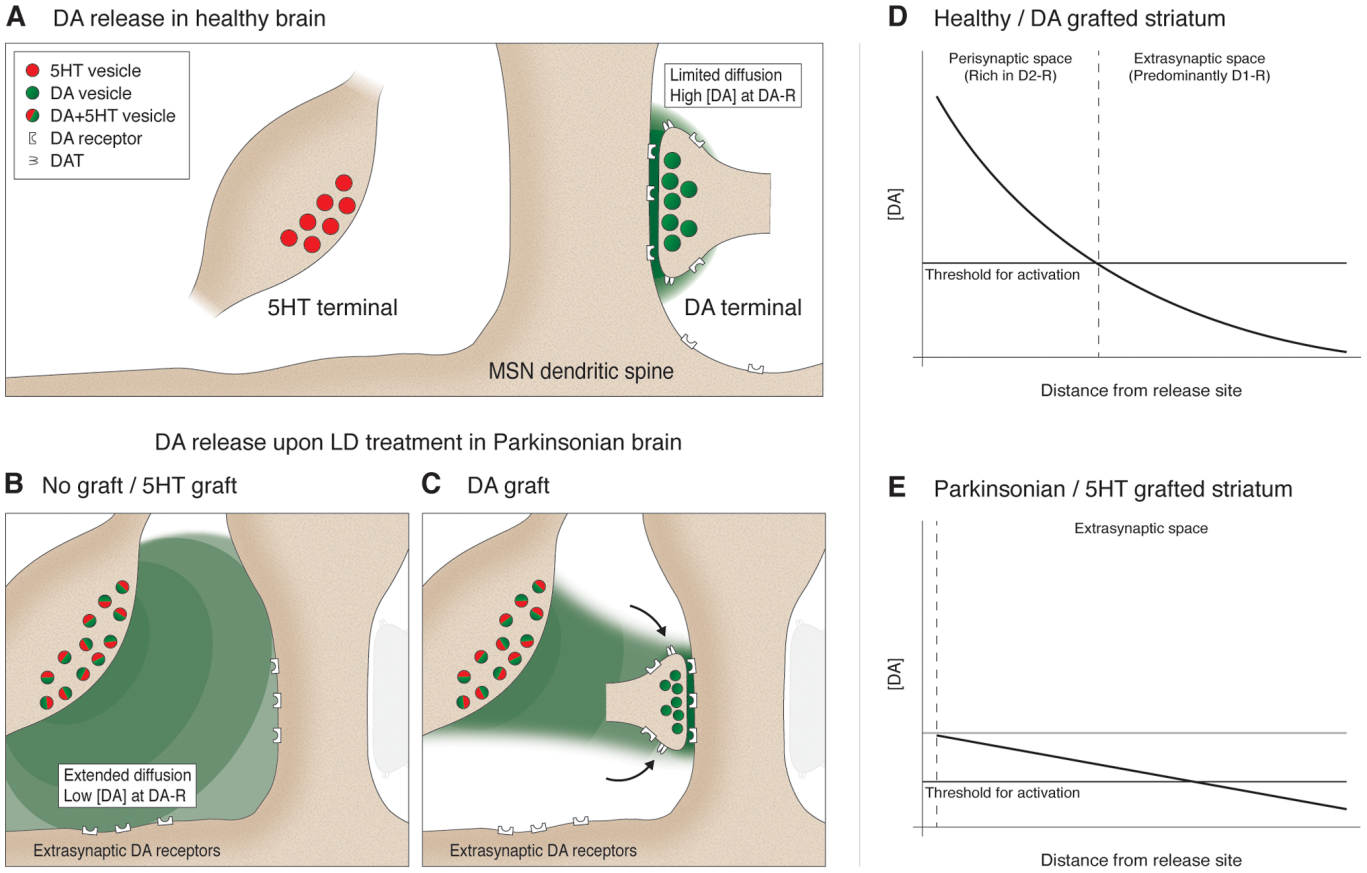
Serotonergic mechanisms for induction of L-DOPA induced dyskinesia in Parkinson's disease. Two distinct properties of dopamine (DA) release from the dopaminergic nerve terminals in the healthy brain (A) distinguish it from the dopaminergic neurotransmission seen in Parkinson's disease (PD) (B). The ability to release DA at high concentration at the synaptic site and control the spread by way of the uptake sites creates a sharp gradient in DA concentration as a function of distance providing selectivity in the receptors activated (D). Extrasynaptic DA-receptors are typically activated only after burst discharges (not illustrated in this figure). Lack of appropriate synaptic specializations at the correct target locations and the absence of re-uptake sites for DA makes the serotonergic nerve terminals behave differently upon L-DOPA administration (B). DA released from these terminals as a false neurotransmitter results in not only a wider diffusion but also fails to create the selectivity in the activation pattern leading to an abnormal dopaminergic signaling (E). Note that under these circumstances the threshold for activation of receptors may also be altered. Nevertheless, dopamine-neuron rich grafts re-establish the proper synaptic release mechanisms, limit the diffusion of DA in the extracellular space and therefore restore physiological neurotransmission (C, D).

Our current working hypothesis (illustrated in Fig. 10) proposes the following mechanism: In the intact brain or the DA grafted striatum (Fig. 10A, C), DA release from the appropriate sites results in a sharp rise in DA concentrations at the release site, followed by a rapid clearance via the DAT, thus creating a limited sphere of influence with a sharp concentration gradient as a function of distance and a brief time window of activation after each release event (Fig. 10D), providing a temporal and spatial selectivity to the physiological neurotransmission [47]–[49]. It follows that, under normal conditions, extra-synaptic D1R are activated selectively after burst discharges [49], whereas, DA released from 5HT terminals would neither create sufficiently high concentrations at D2R containing sites, nor would it be able to establish a clearance mechanism (i.e., lack selectivity in the activation pattern) (Fig. 10B). DA release from compartments that do not contain functional DAT causes DA to persist in the extracellular space much longer [50], [51]. This would cause DA to diffuse much further in the extracellular space and act on the D1R with a wider sphere of influence (Fig. 10E). Such abnormal activation of D1R by L-DOPA has been linked to abnormal internalization of D1R in the striatum [38], [52], [53]. On the other hand, restoration of a new DA-neuron based terminal release network would establish an appropriate synaptic release mechanism, normalize the D2R occupancy, control the leakage of DA in the extra-synaptic space, and thus also restore the normal activation pattern of D1R (Fig. 10C).

A logical interpretation of this outcome can be based on the differential ultrastructural properties of the two types of terminals. DA neurons are known to innervate their striatal target cells at very high intensity forming not only synaptic contacts but also a dense axon lattice. Detailed electron microscopy studies investigating dopaminergic synapses demonstrated that majority of pre-synaptic DA terminals either make synaptic contacts with the spine neck of the striatal target neurons or reside in close proximity to these specialized sites [54]–[56]. By contrast, serotonergic terminals make proper synaptic contacts infrequently. Instead 5HT release typically occurs from varicosities located *en-passant* to the 5HT receptor baring sites[57]. Therefore it is plausible that the serotonergic terminal network – either endogenous or those established by the grafted neurons – release DA at sites further away from the release sites normally generated by DA neurons. As a consequence, while DA may be released in an uncontrolled manner from serotonergic terminals and diffuse further due to lack of uptake sites, it fails to reach sufficient concentration locally at the D2R containing sites. In support of this view, the insufficiency of the DA released from serotonergic terminals as compared with that originating from the dopaminergic neurons became evident in our OMD experiments upon blockade of the re-uptake sites with nomifensine.

DA terminals provide both continuous dopaminergic stimulation and a buffering capacity in the areas they innervate. It is likely that this is the underlying mechanism by which dopaminergic grafts reduce dyskinesia and abolish an abnormal response pattern of the striatal neurons [10], [58]–[60]. Normalization of δFosB expression after reconstitution of the appropriate DA release sites is an important indicator of this difference [61]. Here we demonstrated both retention of abnormal accumulation of δFosB and a concomitant persistence of dyskinesia in both the DA denervated rats and the 5HT-grafted animals. The relationship between ΔFosB and dyskinesia has been well documented [7], [62]–[64] and reduction of δFosB expression by antisense oligonucleotide treatment has been shown to reduce the severity of dyskinesia in rats [62]. Moreover, striatal δFosB induction occurs in dynorphin containing projection neurons of the direct pathway and is mediated by D1R activation [7], [37], [65].

The pre-synaptic dopamine-releasing compartment has a strong impact on the behavior of the post-synaptic striatal neurons. When striatal dopamine production is inhibited by knockdown of the TH enzyme – causing a functional DA depletion without a structural disintegration of the synaptic terminals – pulsatile administration of exogenous L-DOPA fails to induce dyskinesia [26]. Under these experimental conditions, even when dyskinesia is induced by direct DA-receptor stimulation by chronic apomorphine treatment and striatal FosB/δFosB expression is elevated, re-constitution of substantial DA neurotransmission from endogenous terminals upon L-DOPA supplement fails to elicit dyskinesia. These results support the view that DA released from proper synaptic contacts with appropriate auto-receptor control mechanisms and re-uptake sites introduces very low or no risk for induction of dyskinesia in rats, despite providing higher levels of DA release than achieved via serotonergic terminals.

Taken together, our results suggest that in cases where dyskinesia is troublesome and there is an abundance of serotonergic terminals in areas of the striatum lacking dopamine terminals, the side effects of L-DOPA are induced not because of excessive DA production but rather a limited dopaminergic stimulation primarily targeting extra-synaptic receptor sites that persists only intermittently. In other words, the abnormality of DA release from serotonin neurons not only has a quantitative insufficiency, but also a qualitative abnormality, such as lack of appropriate gradients from release sites, specificity of activation in subsets of receptors needed to mediate appropriate actions and inability to buffer the extracellular levels of DA to mediate normal neurotransmission. A therapeutic intervention dampening DA release from the serotonergic terminals might be effective in reducing dyskinesia, however in order to achieve substantial functional restoration in these cases, it would be necessary to provide an additional pool of DA that can normalize the activation pattern of DA receptors (increase occupancy at D2R and reduce activation of extrasynaptic D1R) and sustain this activity over a long term, e.g., by enriching the dopaminergic terminal density in denervated regions of striatum especially in areas that receive 5HT innervation.
